# Patients' opinion on the use of 2 generations of power‐driven water flossers and their impact on gingival inflammation

**DOI:** 10.1002/cre2.456

**Published:** 2021-05-31

**Authors:** Kristina Bertl, Pia Edlund Johansson, Andreas Stavropoulos

**Affiliations:** ^1^ Department of Periodontology, Faculty of Odontology University of Malmö Malmö Sweden; ^2^ Division of Oral Surgery University Clinic of Dentistry, Medical University of Vienna Vienna Austria; ^3^ Division of Conservative Dentistry and Periodontology University Clinic of Dentistry, Medical University of Vienna Vienna Austria; ^4^ Division of Regenerative Dental Medicine and Periodontology University Clinics of Dental Medicine (CUMD), University of Geneva Geneva Switzerland

**Keywords:** AirFloss, bleeding on probing, interdental cleaning device, patients' opinion, periodontitis, questionnaire

## Abstract

**Objectives:**

To assess patients' opinion on the use of 2 generations of power‐driven water flossers and their impact on gingival inflammation.

**Material & Methods:**

In the present prospective cohort study 24 periodontitis patients under regular supportive periodontal therapy used daily 2 generations of a power‐driven water flosser (Sonicare AirFloss [SAF] and Sonicare AirFloss Ultra [SAFU]) for 12 weeks each. Patients were instructed to position the nozzle interproximally from the buccal aspect at each interproximal space. Patients' opinion was assessed by a questionnaire and interproximal bleeding on probing (BoP) was recorded.

**Results:**

Overall satisfaction with SAF/SAFU was rated high, by about 80% of the patients. About 66% of the patients preferred SAF/SAFU compared to their previous interdental cleaning device and indicated that they would continue using SAF/SAFU after the study; none of the patients reported any discomfort or pain. Compared to only tooth brushing, daily use of SAF/SAFU caused a significant reduction of interproximal BoP values, which were well maintained over 6 months; that is, BoP at interproximal buccal and oral sites (pooled), as well as at interproximal buccal and oral sites separately, was proportionately reduced by 29.1%, 41.2%, and 24.8%, respectively (pooled: *p* = 0.027; buccal sites: *p* = 0.030; oral sites: *p* = 0.030).

**Conclusion:**

Patients were very fond of the power‐driven water flossers tested herein, and daily use of the devices for 6 months (i.e., each device was used for 3 months) resulted in a significant reduction of gingival inflammation interproximally.

## 1 BACKGROUND

To achieve sufficient oral hygiene with low plaque and bleeding values, a manual or electric toothbrush should be combined with an interdental cleaning device; if only a toothbrush is used, relatively high amounts (i.e., almost 60%) of plaque remain on the tooth surfaces (van der Weijden & Slot, 2011, 2015). Interdental brushes should be the first choice, unless they cannot be used without traumatizing the interdental tissues (Chapple et al., 2015; Sälzer et al., 2015), but dental floss is still frequently recommended by dentists. In this context, patients often do not follow recommendations for interdental cleaning; that is, irrespective of patient age only about 1/3 flosses once a day, and even less are actually flossing in an acceptable way (Lang et al., 1995; Srinivasan et al., 2019; Winterfeld et al., 2015). In fact, in a survey among >2000 U.S. adults, 27% tend to lie to their dentists about their flossing habits and 36% would prefer doing other unpleasant activities over daily flossing (e.g., cleaning the toilet, working on the taxes, or washing a sink full of dirty dishes) (American Academy of Periodontology, 2019). In perspective, although interdental brushes are likely more accepted than flossing (Christou et al., 1998), it still appears very relevant to obtain more efficient, easy‐to‐use interdental cleaning devices, such as power‐driven interdental cleaning devices.

Power‐driven water flossers have been introduced into the market almost 60 years ago and although their plaque removal efficacy is discussed controversially, it is rather consistently reported that they lower bleeding and gingivitis indices, even if plaque levels are not affected (Husseini et al., 2008; Kotsakis et al., 2018; Worthington et al., 2019); however, one should keep in mind, that information on plaque values is often not given in detail (i.e., the region of plaque assessment [full‐mouth vs. buccal/oral vs. interdental] is not clearly taken into account in these systematic reviews). Specifically, a recent network meta‐analysis (Kotsakis et al., 2018) showed power‐driven water flossers as second most effective adjunct to tooth brushing after interdental brushes, in terms of reducing gingival inflammation. Several theories are discussed about how power‐driven water flossers are able to lower the bleeding indices, despite a non‐significant effect on plaque levels; for example, by altering the biofilm composition, interfering with plaque maturation, stimulating an immune response, reducing the thickness of the plaque, etc. (for overview see Husseini et al., 2008). A more recently introduced power‐driven interdental cleaning device is the AirFloss with a specific air and micro‐droplet technology; that is, the Sonicare AirFloss (SAF) and in its second generation the Sonicare AirFloss Ultra (SAFU) (Royal Philips N.V., Amsterdam, the Netherlands). The mechanism of the SAF/SAFU differs from other power‐driven water flossers (e.g., oral irrigators) in some important aspects; that is, while oral irrigators use a jet stream of water at low velocity, SAF/SAFU emits a microburst of high velocity air and liquid micro‐droplets, causing a shear stress on the tooth surface to detach any biofilm accumulation. There is only limited data on the clinical efficacy or patient related outcome parameters of SAF/SAFU use; these data are limited to periodontally healthy participants or gingivitis patients after single or short‐term use (i.e., maximum of 4 weeks) (Goyal et al., 2015; Heiß‐Kisielewsky et al., 2015; Mwatha et al., 2017; Sharma et al., 2012a, 2012b; Stauff et al., 2018).

Thus, the aim of the present study was to assess patients' opinion on the use of 2 generations of power‐driven water flossers and their impact on gingival inflammation among periodontitis patients under supportive periodontal therapy for 6 months.

## 2 MATERIAL AND METHODS

### 2.1 Patient population

The present prospective cohort study was approved by the Ethics Committee at Lund University (Lund, Sweden; DNR 2014/388) and reporting complies with the STROBE guidelines (Table S1). The patient population assessed herein is identical with the population of a previous publication, where eligibility criteria have been described in detail (Bertl, Geissberger, et al., 2021). Shortly, 24 periodontitis patients, who were at the timepoint of enrolment already scheduled to supportive periodontal therapy every 3 to 4 months, were included herein, provided that they had: (a) a minimum of 20 teeth present; (b) ≥1 interproximal space between premolars or molars per quadrant; (c) ≥ 5 residual interproximal periodontal pockets (i.e., ≥4 mm with bleeding on probing [BoP]) within the whole dentition; (d) no antibiotic intake in the last 3 months; (e) no medication intake related to gingival hyperplasia; and (f) no pregnancy.

### 2.2 Study outline

After the patients agreed to participate, patients were instructed not to use any interdental cleaning device 1 week prior to a baseline appointment. At this baseline appointment, bleeding index was recorded, and each participant received a brand‐new SAF device (Royal Philips N.V., Amsterdam, the Netherlands) including detailed instructions about how to use it. Patients' habits, in terms of manual or electric toothbrush were not changed, that is, the patients continued brushing as prior to study entrance. After 12 weeks the participants returned, bleeding index was recorded again, and a questionnaire was given to each participant. Then, the participants received a brand‐new SAFU device (Royal Philips N.V., Amsterdam, the Netherlands) including instructions how to use it. Due to the primary aim of the present study (Bertl, Geissberger, et al., 2021) assessing whether bacterial colonization in a power‐driven water flosser can be prevented by using SAF/SAFU in different ways (i.e., with bottled water or with an essential‐oils [EO]‐based mouth‐rinse), a 1‐week wash‐out period was scheduled between the SAF and SAFU test trial and participants were again instructed not to use any interdental cleaning device within this 1 week prior to using SAFU. After another 12 weeks the participants returned, bleeding index was recorded for the last time, and the same questionnaire was given a second time to each participant. Thus, the regular 3‐monthly periodontal supportive therapy was not disrupted.

### 2.3 Use of the SAF/SAFU

At baseline, participants were instructed to use the SAF/SAFU once per day after tooth‐brushing (either morning or evening), by positioning the tip of the nozzle from the buccal aspect at each interproximal space and pressing the button once; thus, SAF delivered a single burst, while SAFU was pre‐set to deliver a triple burst. During the study period the participants were asked not to use any other interdental cleaning device or mouth‐rinse. Based on the primary aim of the project (i.e., assessing any possible bacterial colonization within the SAF/SAFU), the participants were randomly allocated to a specific way of use (for details see Bertl, Geissberger, et al., 2021). Furthermore, patients were instructed not to use the device 24 h before returning to the clinic. In the present study, only potential differences between those participants using SAF intra‐orally with bottled water (1 bottle/week; Evian®, Malmö, Sweden) or with an EO‐based mouth‐rinse (Listerine® Total Care; Johnson & Johnson Consumer Nordic) were considered as relevant.

### 2.4 Questionnaire on patients' opinion

At the end of each 12‐week trial, the patients received a questionnaire asking about their opinion on the SAF/SAFU. These questions included: (a) presence, intensity, and location of pain; (b) satisfaction on a scale from 1 to 10 (i.e., score 1 representing “very satisfied,” and score 10 “very dissatisfied”) with regards ease of use, slip resistance of the grip, accessibility of the interproximal sites of the posterior teeth, and cleaning capacity; (c) overall satisfaction on a scale from 1 to 10; (d) information on type and frequency of use of previous interdental cleaning devices; (e) preference when comparing SAF/SAFU with previously used interdental cleaning devices; (f) willingness to take SAF/SAFU for traveling, to buy it, to recommend it to friends/family members, and to continue using it after the study; g) price willing to pay; and h) any additional comments.

### 2.5 Impact on gingival inflammation

Secondary aim of the study was to assess efficacy of the SAF/SAFU over a 6‐month study period. Participants were instructed not to use any interdental cleaning device 1 week prior to the baseline appointment, that is, prior to combining toothbrushing with SAF/SAFU on a daily base. As index for gingival inflammation BoP was recorded by a single, previously calibrated examiner (PEJ) at baseline, after 12 weeks, and at the end of the trial (i.e., after 6 months). BoP was assessed interproximally at all first and second premolars and molars; that is, at the disto‐buccal/‐oral and at the mesio‐buccal/‐oral sites, excluding the mesial aspect of the first premolar and the distal aspect of the second molar, thus a maximum of 48 sites per patient were included. Presence/absence of BoP was assessed 20 to 30 s after regular periodontal probing with approximately 0.2 N and a standard periodontal probe; BoP was expressed in percent of sites positive for bleeding out of the total number of included sites: (a) for all sites (i.e., buccal and oral sites pooled) and (b) separately for buccal and oral sites.

### 2.6 Statistical analysis

The data derived from the questionnaires were descriptively summarized (i.e., frequency and distribution) and for those questions with a scale from 1 to 10, scores 1 to 3 were considered as patients being in general satisfied, scores 4 to 7 as patients being moderately satisfied, and scores 8 to 10 as being dissatisfied. Any differences in patients' opinion comparing SAF and SAFU were assessed by McNemar or McNemar‐Bowker test and any differences in BoP comparing baseline and follow‐up after using SAF and SAFU were assessed by Wilcoxon‐signed‐ranks test; that is, both approaches are for dependent data. The comparison between individuals using the SAF with water versus those using it with an EO‐based mouth‐rinse was performed with the Mann–Whitney‐U‐test. A statistical program (SPSS, Version 24.0, Chicago, IL, USA) was used and a *p*‐value < 0.05 was considered statistically significant.

## 3 RESULTS

### 3.1 Patient population

No remarkable events occurred during the first 12‐week experimental period regarding the use of SAF. Out of the 24 patients (14 female, 10 male; age range: 32 to 73 years, mean age: 52.9 ± 11.2 years), 23 patients filled in the questionnaire and BoP was recorded for 22 patients (2 patients returned only the device in time but canceled the clinical assessment and only one of them filled in the questionnaire). Two patients dropped out of the study after using the SAF (1 patient did not return, and 1 patient started with orthodontic treatment). Additionally, several technical complications occurred during the second 12‐week experimental period, regarding the use of SAFU; that is, in several cases the device stopped working due to a defect battery during the 12‐week period. Altogether, 16 patients used the SAFU for 12 weeks as instructed and BoP was recorded; 2 additional patients, whose device stopped functioning 1 to 2 weeks prior to the clinical assessment, filled in the questionnaire resulting in 18 answers in total for the SAFU, but their clinical data were not included.

### 3.2 Patients' opinion

**FIGURE 1:**
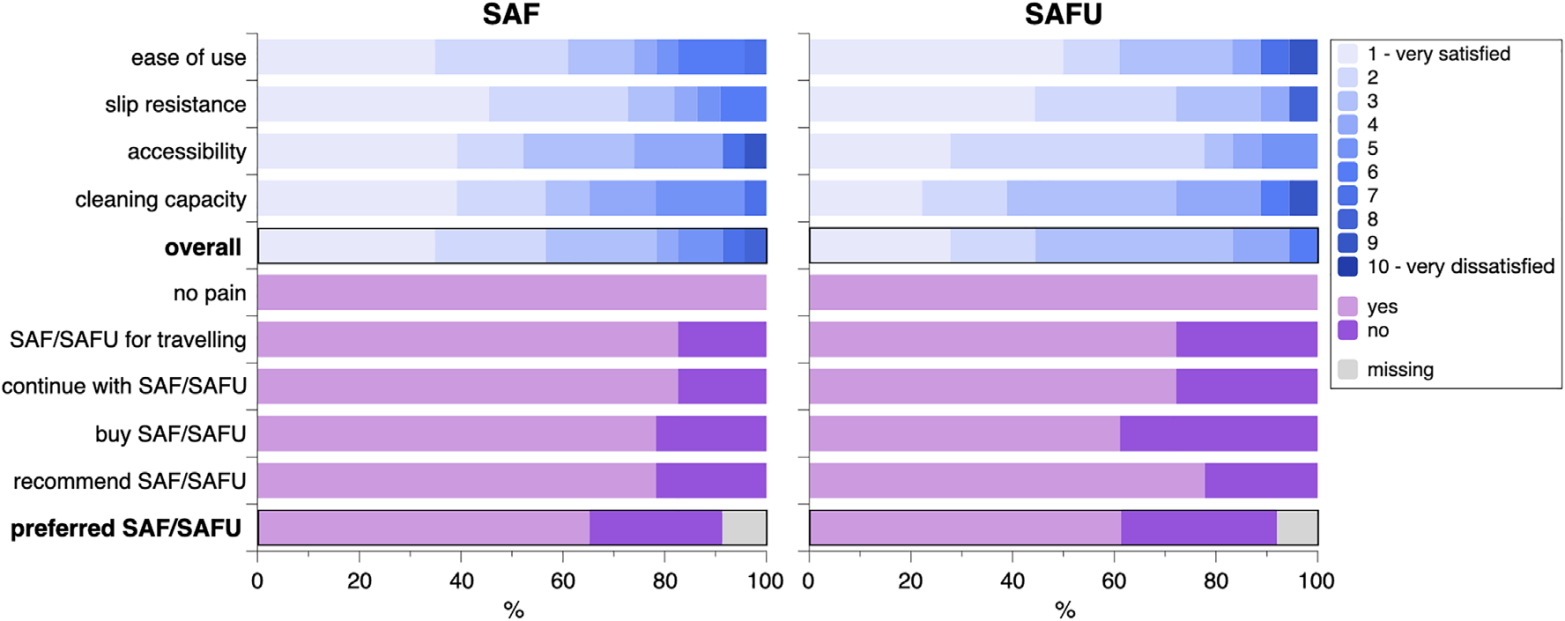
Patients' opinion on the SAF and SAFU

No patient experienced pain or any other remarkable adverse event while using SAF/SAFU. Patients' satisfaction with ease of use, slip resistance of the grip, accessibility of the interproximal sites of the posterior teeth, and cleaning capacity was judged high; that is, 65.2% to 81.8% and 72.2% to 88.9% of the patients reported for the SAF and SAFU, respectively, to be in general satisfied (i.e., indicated by a score of 1, 2, or 3). Further, the overall satisfaction with the SAF and SAFU was rated high (i.e., achieved a score of 1, 2, or 3) by 78.3% and 83.3% of the patients, respectively. More than 70% of the patients would use SAF/SAFU also during traveling and more than 75% of the patients would recommend it to friends/family members. All participants had been using an interdental cleaning device prior to entering this study; 56.5% had been using interdental brushes, 34.8% a combination including interdental brushes, and 4.3% each floss alone or a combination not including interdental brushes. Further, 73.9% had been using the previous interdental cleaning device daily, 17.4% at least 4‐times per week, and 8.6% ≤3‐times per week. About two thirds of the patients preferred SAF/SAFU compared to their previous interdental cleaning device and stated that they would continue using it after the study; patient would pay up to ca. 100 € for the device. However, a few patients indicated among the general comments, that the cleaning function might be inferior to interdental brushes. The patient related outcomes are summarized in Figure 1; none of these outcomes presented a statistically significant difference between SAF and SAFU (*p* ≥ 0.375).

### 3.3 Impact on gingival inflammation

**FIGURE 2:**
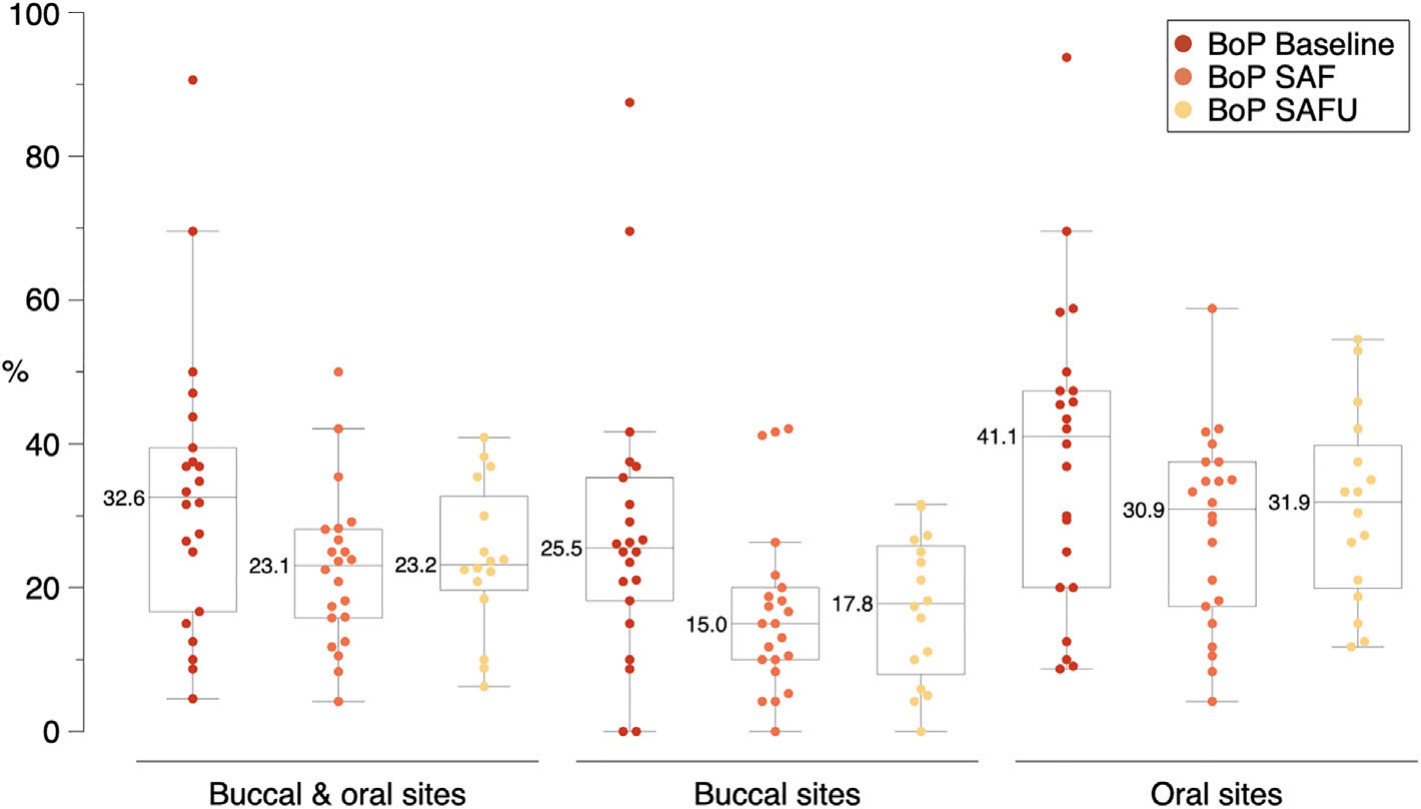
Interproximal bleeding on probing (BoP) values at baseline and after using SAF and SAFU for 12 weeks each (i.e., 6 months in total) at interproximal buccal and oral sites pooled, as well as at interproximal buccal and oral sites separately

After 1 week without using any interdental cleaning device the interproximal BoP values were 32.6%, which was significantly reduced until the end of the first 12‐week trial to 23.1%. Specifically, BoP at interproximal buccal and oral sites (pooled) as well as at interproximal buccal and oral sites separately was reduced by about 10 units, proportionately corresponding to a reduction of 29.1%, 41.2%, and 24.8%, respectively (pooled: *p* = 0.027; buccal sites: *p* = 0.030; oral sites: *p* = 0.030) (Figure 2). These BoP values remained stable (within a range of ±3% in the BoP value) after using the SAFU for another 12 weeks until the end of the study.

No differences were detected between participants using SAF with water (*n* = 15) versus those using it with an EO‐based mouth‐rinse (*n* = 7) regarding BoP values at the end of the 12‐week trial (*p* ≥ 0.123, data not shown), nor regarding the changes of BoP values from baseline to the end of the 12‐week trial (*p* ≥ 0.490, data not shown). Such a comparison was not possible for the SAFU, as the clinical data of only a single participant using it with an EO‐based mouth‐rinse were available.

## 4 DISCUSSION

Cleaning of the interproximal space is not sufficient with a toothbrush only and patients should use an interdental cleaning device. However, although flossing is frequently recommended by dentists, it is neither easy‐to‐use nor well accepted by most patients (American Academy of Periodontology, 2019). SAF/SAFU are power‐driven interdental cleaning devices, which appear easy to use and thereby potentially achieve a high acceptance among patients. The present study summarized patients' opinions about SAF/SAFU after regular use for 6 months. Indeed, herein the overall satisfaction with SAF/SAFU was rated high by about 80% of the patients and none reported any discomfort or pain while using the device; that is, the pressure of the water burst (i.e., single and triple burst) appears well accepted by the patients. Further, more than 75% of the patients would take SAF/SAFU for traveling and they would recommend the device to family members and friends. The present study included periodontitis patients, who had been primarily using interdental brushes prior to entering the study (i.e., >90% of the cases); about 2/3 of the patients preferred SAF/SAFU compared to their previous interdental cleaning device and indicated that they would continue using it after the study. Similarly, in a previous study (Heiß‐Kisielewsky et al., 2015) assessing the preference of dental students after a single use of SAF, comparably high values in favor of SAF were reported; specifically, 82% of the students judged the device as easy‐to‐use and 59% preferred it compared to flossing. One may thus consider that such devices may be advantageous for patients, who are less motivated/compliant or have inferior dexterity and/or difficulty in handling other type of interdental cleaning devices.

In this context, 95% of the students in the above‐mentioned study (Heiß‐Kisielewsky et al., 2015), judged flossing as more effective. In another study, gingivitis patients were also in favor of flossing in terms of self‐reported effectiveness (Stauff et al., 2018). Herein, about 65%–70% of the patients were in general satisfied with the cleaning capacity of SAF/SAFU, but some of the general comments in the questionnaire indicated that at least some of the patients felt somehow insecure about the cleaning efficacy of SAF/SAFU (e.g., “interdental brushes are more effective,” “I would use it only additionally to other IDB devices,” “it might work better if one has healthy and not malpositioned teeth,” etc.). However, controversial results have been obtained in laboratory studies on the cleaning efficacy of SAF/SAFU. Specifically, while in vitro biofilm tests presented a promising reduction of the biofilm thickness after exposure to the microburst of high velocity air and liquid micro‐droplets (Fabbri et al., 2016a, 2016b; Rmaile et al., 2015), other studies reported SAF/SAFU as inferior to remove surface coatings/simulated biofilms compared to flossing or interdental brushes (Holley et al., 2014; Tuna et al., 2019). Similarly controversial are the results of the few clinical studies, published up‐to‐now in peer‐reviewed journals. Specifically, in terms of plaque reduction comparable (Mwatha et al., 2017; Stauff et al., 2018) as well as inferior (Heiß‐Kisielewsky et al., 2015) results of SAF/SAFU compared to flossing are reported. For example, when gingivitis patients with irregular interdental cleaning habits at timepoint of recruitment used either SAFU or floss for a 4‐week period, a significant reduction in plaque accumulation and bleeding, but without significant inter‐group differences was observed (Stauff et al., 2018). Due to the primary aim of the present study (i.e., assessment of bacterial colonization in SAF/SAFU, Bertl, Geissberger, et al., 2021) the participants were instructed not to use the device 24 h before returning it to the clinic; since it is expected that some plaque would have accumulated interproximally after >24 h, an interproximal plaque index was deemed meaningless to assess herein.

However, SAF/SAFU might be clinically efficient in a similar way as reported for oral irrigators; that is, bleeding indices are reduced although plaque reduction is inferior compared to other interdental cleaning devices (Husseini et al., 2008). Indeed, herein a clinically relevant reduction in BoP from baseline to the end of each 12‐week period with SAF and SAFU was observed; that is, the reduction was well maintained over 6 months. Specifically, BoP at the interproximal buccal and oral sites pooled as well as at the interproximal buccal, and oral sites separately was proportionately reduced by 29.1%, 41.2%, and 24.8%, respectively, after using SAF for 12 weeks and these values remained stable while using SAFU for another 12 weeks. As mentioned earlier, in the present study patients refrained from using any interproximal cleaning device 1 week prior to study entry, in order to somehow make the “abandoning” of the previous means of interproximal cleaning and the transition to the new device easier for the participants. It is reasonable to assume that absence of interdental cleaning for 1 week, would have only slightly/insignificantly negatively influenced BoP values at baseline, and that the observed significant reduction in BoP indicates the clinical efficacy of SAF. This view is also supported by the results of a recently published 6‐year prospective study, reporting on the benefits of oral irrigators in terms of periodontitis recurrence during supportive periodontal therapy (Costa et al., 2020). Specifically, it was shown that interdental brushes or oral irrigators as adjuncts to brushing and flossing reduced the rate of periodontitis recurrence by 10% and 20%, respectively, compared to brushing and flossing only; unfortunately, the type of oral irrigator was not further specified therein. In this context, studies comparing specifically oral irrigators with SAF/SAFU are rare, but up‐to‐now favor oral irrigators; however, all these studies were sponsored from the company producing the oral irrigators (Goyal et al., 2015; Sharma et al., 2012a, 2012b). Further, the present study does not allow proper assessment of the clinical efficacy of SAF/SAFU due to limitations like the lack of full‐mouth data, lack of plaque values, and lack of a randomized cross‐over design (i.e., all patients started with the SAF device instead of starting randomly either with SAF or SAFU). Hence, future, appropriate designed, ideally industry‐independent trials, assessing the clinical efficacy of SAF/SAFU in different patient groups (i.e., orthodontic patients, healthy individuals, gingivitis patients, periodontitis patients, etc.) and with different ways of use (i.e., water versus mouth wash, buccal versus lingual positioning of the nozzle, etc.) are required for better understanding of the clinical efficacy and limitations of SAF/SAFU. At this point, it has to be pointed out that these devices have the limitation of bacterial colonization of the nozzle and the device itself (Bertl, Geissberger, et al., 2021; Bertl, Johansson, et al. 2021), and patients should be advised not to share the device with family members/partners.

In conclusion, the present data show that about 80% of the patients are overall very satisfied with SAF/SAFU, and about 2/3 of the patients preferred SAF/SAFU compared to their previous interdental cleaning device. Further, interproximal BoP values were significantly reduced and well maintained for 6 months.

## CONFLICT OF INTEREST

The authors declare no conflict of interest.

## AUTHOR CONTRIBUTIONS

*Study idea, data analysis, data interpretation, manuscript drafting*: Kristina Bertl, Andreas Stavropoulos. *Clinical procedures, study administration, manuscript revision*: Pia Edlund Johansson.

## Supporting information


**Table S1.** STROBE Statement—Checklist of items that should be included in reports of cohort studies.

## Data Availability

Data available upon reasonable request.
